# Role of perioperative nutritional status and enteral nutrition in predicting and preventing post-operative complications in patients with Crohn’s disease

**DOI:** 10.3389/fnut.2022.1085037

**Published:** 2023-01-06

**Authors:** Tianyu Jiang, Yongmei Jiang, Qianwen Jin, Shining Xu, Abraham Fingerhut, Yongmei Shi, Minhua Zheng, Zirui He

**Affiliations:** ^1^Department of General Surgery, School of Medicine, Ruijin Hospital, Shanghai Jiao Tong University, Shanghai, China; ^2^Shanghai Minimally Invasive Surgery Center, Shanghai, China; ^3^Department of Clinical Nutrition, School of Medicine, Ruijin Hospital, Shanghai Jiao Tong University, Shanghai, China; ^4^Department of Surgery, Section for Surgical Research, Medical University of Graz, Graz, Austria

**Keywords:** Crohn’s disease, nutritional assessment, oral supplement, enteral nutrition, post-operative complications

## Abstract

**Background:**

Perioperative immune-nutritional status is correlated with post-operative outcomes. This study aimed to evaluate whether pre-operative nutritional status could predict post-operative complications in patients with Crohn’s disease (CD) and whether pre-operative enteral nutrition (EN) can prevent post-operative complications.

**Methods:**

This retrospective cohort study analyzed the electronic health records of 173 patients diagnosed with CD in Ruijin Hospital, Shanghai, China, between August 2015 and May 2021: 122 patients had pre-operative nutritional support while 51 patients underwent surgery without pre-operative nutritional support. The pre-operative nutritional status, disease activity index, disease-related data, frequency of multiple surgery, operative data, and post-operative characters in each group were compared to determine whether the nutritional support and status could significantly affect post-operative outcome. One-to-one propensity score matching (PSM) was performed to limit demographic inequalities between the two groups.

**Results:**

After PSM, no statistically significant differences were found in pre-operative patient basic characteristics between the two groups of 47 patients (98 patients in all) included in this study. Overall, 21 patients developed 26 post-operative complications. In terms of pre-operative nutritional status, the level of serum albumin (ALB), pre-albumin (pre-ALB), and hemoglobin (Hb) in the nutrition group were statistically higher than that in the control group. We also observed a statistically significant decrease in post-operative complications, need for emergency surgery, and staged operations, while the rate of laparoscopic surgery was higher in the nutrition group compared to the non-nutritional group. Post-operative complications were related to pre-operative nutritional condition, which indicated that EN may improve the nutritional status and reduced the rate of post-operative complications.

**Conclusion:**

Pre-operative nutritional status is correlated with post-operative outcomes while EN plays a positive role in preventing the post-operative complications. EN is useful for improving the pre-operative nutritional status and reducing the post-operative adverse events for CD patients undergoing surgery.

## 1. Introduction

Crohn’s disease (CD) is a chronic inflammatory disease of the intestinal mucosa which can occur discontinuously throughout the entire digestive tract. Its pathogenesis is still poorly understood (1, 2). In China, the morbidity and prevalence of CD are increasing constantly, which leads to a heavy economic and social burden (3). Although the innovation and application of series of new drugs has greatly improved the therapeutic effect (4), complications such as intestinal bleeding, obstruction, fistula, perforation, abscess still occur (5, 6), these patients as well as those who respond poorly to conservative treatment may require surgery (7, 8).

During the natural course of CD, the nutritional condition and the diet are important in the etiology of CD, particularly in regions where the rising prevalence rate has paralleled changes in eating habits and food industrialization (9, 10). High prevalence of malnutrition and micronutrient deficiencies were observed in patients with CD and constitutes a challenging issue for patients who require surgery (9). While malnutrition was confirmed to be an independent prognostic factor for increased risk of post-operative complications after abdominal surgery, which contributes to approximate 20–40% of surgical complication rates (11, 12). In this regards, early diagnosis of malnutrition is extremely important since pre-operative nutritional intervention may contribute to lower post-surgical complication and mortality rates (13–15). However, until now, there is few evidence directly linking nutritional status to post-operative complications in CD patients. Evidence that supports the effectiveness of nutritional support for patients with CD during peri-operative period, and whether it can contribute to lower post-operative complication incidence rate is scant (11, 16). Thus, we selected scored Patient-Generated Subjective Global Assessment (PG-SGA score) as a validated tool to screen patients’ immune-nutritional status (17, 18), and we selected the comprehensive complication index (CCI) to quantify the post-operative complications within 30 days.

Furthermore, several strategies were adopted to establish a standard clinical protocol of pre-operative enteral nutrition (EN): including patient nutrition education programs, dietary instruction, partial enteral nutrition (PEN), exclusive enteral nutrition (EEN), and total parenteral nutrition (TPN), which can significantly reduce the effects of catabolism (19). Our primary hypothesis was that pre-operative EN would increase pre-operative immune-nutritional status and reduce 30-day post-operative complications, as measured using the CCI. The objective of this study is to evaluate whether the nutritional status could predict post-operative complications in CD patients and the benefit of EN on the nutritional status and preventing post-operative complications of CD patients.

## 2. Materials and methods

### 2.1. Study population

This retrospective study included all patients undergoing surgery for CD at Ruijin Hospital from 12 August 2015 to 19 May 2021. Included were patients with post-operative histological diagnosis of CD according to the European Crohn’s and Colitis Organization (ECCO) guidelines (7, 8) and Consensus Opinion on Diagnosis and Treatment of inflammatory bowel disease (2018, Beijing) of the Chinese Inflammatory Bowel Disease Group (20), who underwent surgical resection due to failure of medical therapy or developed complications with the following inclusion criteria: (1) age ≥18 years, male or non-pregnant female; (2) American Society of Anesthesiologists (ASA) Grades I–III; and (3) complete follow-up data. All indications were discussed in the multidisciplinary team (MDT) conference including gastrointestinal surgeons, gastroenterologists, nutritionists, pathologists, radiologists, and nurses. Patients with incomplete clinical data or electronic health record were excluded. Patients were divided into two groups according to whether they had received pre-operative nutritional support or not.

This study was approved by the hospital’s Ethics Committee and all patients provided informed consent.

### 2.2. Nutritional assessment

All patients receiving nutritional support were assessed by the PG-SGA score and activity of CD with Crohn’s Disease Activity Index (CDAI) before surgical procedure. The index was assessed by nutritionists and surgeons at the time of admission for surgery and were stored in patients’ clinical database and electronic health record. Patients with CDAI scored 0 to 149 points were identified as remission, while patients with CDAI scored no less than 150 points were identified with active disease, according to the ECCO and Beijing guidelines (7, 8, 20). During the course of nutritional support, nutritional deficiency was screened by blood tests routinely, once every 3 months for patients with mild to severe active disease and once every 6 months in patients in asymptomatic remission. Laboratory data refer to the nutritional condition were collected from electronic health record.

### 2.3. Nutritional support

#### 2.3.1. Protein

Protein requirements for patients in remission was 1 g⋅kg^–1^⋅day^–1^ in adults, similar to that recommended for the general population, while protein requirement was 1.2–1.5 g⋅kg^–1^⋅day^–1^ in adults with active disease (21). All patients were asked to adhere to nutrition education programs. The nutritionist provided individual dietetic recommendation for each patient (22).

#### 2.3.2. Energy

Patients who could not meet the expected energy requirements underwent EN depending on the disease activity, duration of feeding, patient compliance, and gastrointestinal function. For the patients with asymptomatic remission, energy supply was 25–30 kcal⋅kg^–1^⋅day^–1^. PEN was recommended for these patients while 400–1800 kcal⋅d^–1^ through method of EN, the rest of energy requirement was taken by dietary. For patients with active disease, energy supply was 8–10% higher than the remission phase, which was 30–35 kcal⋅kg^–1^⋅day^–1^. For patients whose dietic intake did not meet the standard nutritional requirements, we selected EEN as the method of nutritional support. Patients who could not tolerate EN because of severe obstruction or fistule, TPN was recommended.

#### 2.3.3. Microelement

The supplement of microelements was applied according to the result of laboratory blood test at the discretion of treating physician. Vitamin D and calcium supplements were recommended depending on the assessment of osteoporosis and level of serum calcium when 25-OH-Vit D was inferior to 75 nmol/L. Hypoferric anemia was corrected by iron therapy: oral supplement for the patients of mild iron deficiency anemia (100 g⋅L^–1^ ≤ hb<120 g⋅L^–1^), intravenous iron supplementation for the patients of moderate anemia (hb < 100 g⋅L^–1^). For the loss of vitamin B12, folic acid, potassium, magnesium, calcium, and phosphorus caused by diarrhea in CD patients, oral supplement was applicated according to the results of blood test.

### 2.4. Data collection

Laboratory data including white blood count (WBC), total lymphocyte count (TLC), red blood count (RBC), hemoglobin (Hb), albumin (ALB), pre-albumin (pre-ALB), C-reactive protein (CRP), erythrocyte sedimentation rate (ESR), and parameter such as vitamin D, vitamin K, folic acid, 25-OH-Vit D, serum iron, serum calcium, serum phosphorus, serum magnesium. The prognostic nutritional index (PNI) was calculated from the serum ALB and TLC level, and the formula was PNI = 10 × ALB (g/dL) + 0.005 TLC (per mL).

Intraoperative data including surgical approach (open vs. laparoscopy), surgical option (resection or ostomy), surgical type, staged surgery or not, operation time, estimated blood loss (EBL) and intraoperative mortality or complication.

Post-operative data including post-operative complications recorded up to 30 days after surgery (graded according to the Clavien-Dindo classification), treatment of complications, time to bowel movements, post-operative fluid intake time, and length of stay (LOS) were obtained from electronic medical records. Time to bowel movements, post-operative fluid intake time, total LOS, post-operative LOS, and hospitalization expenses were recorded. CCI integrates all complications with their respective severities on a continuous scale ranging from 0 (no burden due to complications) to 100 (death as a result of complications). The CCI was scored by the CCI calculator available online.^1^

Baseline characteristics data including age, sex, ASA score, body mass index (BMI), comorbidity, smoking and alcohol history, medication history. perioperative data, and laboratory data were collected from the clinical database and electronic health record. CD-related data included CDAI, PG-SGA score, Montreal classification, duration of disease, recurrence rate, and pre-operative drug therapy: either infliximab (documented dose of infliximab for more than 4 weeks before surgery) or corticosteroids (daily dose of 5 mg prednisolone or 4 mg methylprednisolone and within 4 weeks before surgical intervention). Missing data were treated with multiple imputations.

### 2.5. Statistical analysis

All statistical analyses were performed using SPSS 26.0 (Armonk, NY, USA: IBM Corp). PSM analysis was conducted using a logistic regression model with the selected co-variates. We used a caliper width of 0.05 for the pooled standard deviation of the logit for PSM. Demographic and clinical characteristics were summarized and descriptively analyzed, and all quantitative values were presented as means and standard deviations. The Student’s *t*-test or the Mann–Whitney *U*-test and Pearson’s χ^2^ (or Fisher’s exact test) were used to compare continuous and categorical variables, respectively. All values were two tailed, and *p*-values < 0.05 were considered to be significant.

## 3. Results

### 3.1. Patient baseline characteristics

**FIGURE 1:**
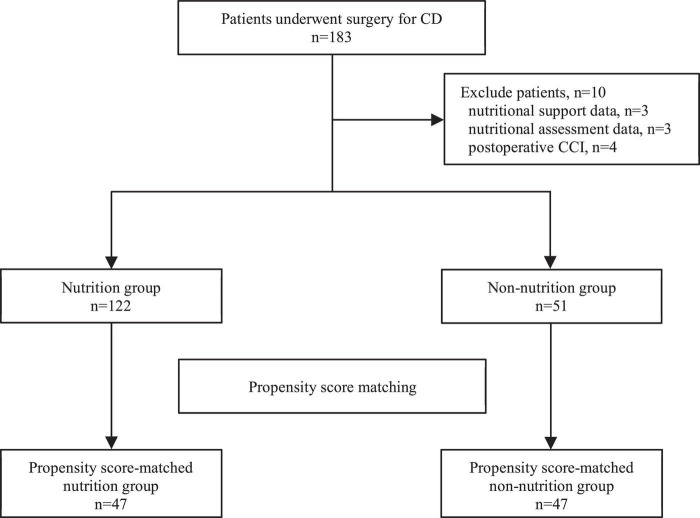
Study flow chat.

The study flow chart was summarized in Figure 1. We identified 183 consecutive cases of CD that underwent surgery from August 2015 to May 2021. We excluded 10 cases due to missing important records: missing nutritional support data (*n* = 3), missing nutritional assessment data (*n* = 3), and missing CCI report (*n* = 4). Hence, 122 cases in the nutrition group and 51 cases in the non-nutrition group were adjusted by PSM. Table 1 shows the baseline characteristics of the 173 patients. Compared with patients in the non-nutrition group, patients in nutrition group had younger ages (*p* = 0.031), higher ASA score (*p* = 0.002). The PSM analysis was conducted using a logistic regression model with the following co-variates: age, sex, ASA Score, and drug therapy (Table 1).

**TABLE 1:** Patient demographics characteristics.

	Before PSM		*P*-value	After PSM		*P*-value
	Nutrition	Non-nutrition		Nutrition	Non-nutrition	
No. of patients (%)	122 (70.5)	51 (29.5)		47 (50)	47 (50)	
Age (years)	37.9 ± 11.9	42.5 ± 14.5	**0.031**	39.8 ± 13.0	40.3 ± 12.5	0.865
Gender (%)			0.459			0.652
Male	79 (64.8)	36 (70.6)		34 (72.3)	32 (68.1)	
Female	43 (35.2)	15 (29.4)		13 (27.7)	15 (31.9)	
BMI	19.59 ± 3.32	20.01 ± 3.08	0.448	19.08 ± 3.43	20.01 ± 3.15	0.18
ASA score (%)			**0.002**			0.876
I/II	51 (41.8)	8 (15.7)		44 (93.6)	43 (91.5)	
III/IV	71 (58.2)	43 (84.3)		3 (6.4)	4 (8.5)	
Duration of disease (months)	63.59 ± 55.32	54.33 ± 65.90	0.414	75.70 ± 50.00	58.69 ± 67.48	0.264
**Montreal classification**						
Age (%)			0.067			0.816
A1	3 (0.6)	1 (1.7)		1 (2.1)	1 (2.1)	
A2	84 (48.6)	26 (15.0)		29 (61.7)	26 (55.3)	
A3	35 (20.2)	24 (13.9)		17 (36.2)	20 (42.6)	
Location (%)			0.083			0.161
L1	40 (23.1)	22 (12.7)		17 (36.2)	21 (44.7)	
L2	15 (8.7)	7 (4.0)		4 (8.5)	7 (14.9)	
L3	63 (36.4)	17 (9.8)		24 (51.1)	14 (29.8)	
L1 + L4	4 (2.9)	5 (2.3)		2 (4.3)	5 (10.6)	
B (%)			0.488			0.733
B1	2 (1.2)	1 (0.6)		2 (4.3)	1 (2.1)	
B2	69 (40.1)	24 (14.0)		24 (51.1)	22 (46.8)	
B3	50 (29.1)	26 (15.1)		21 (44.7)	24 (51.1)	
Drug therapy (%)			0.183			0.298
Monoclonal antibody	28 (16.2)	9 (5.2)		12 (25.5)	9 (19.1)	
Immunosuppressor	35 (20.2)	9 (5.2)		14 (29.8)	8 (17.0)	
5-ASA	28 (16.2)	11 (6.4)		11 (23.4)	11 (23.4)	
Steroids	9 (5.2)	5 (2.9)		3 (6.4)	5 (10.6)	
None	22 (12.7)	17 (9.8)		7 (14.9)	14 (29.8)	

The bold values represent *p* < 0.05.

### 3.2. Patient demographics characteristics

After 1:1 PSM, there were 47 patients in each treatment strategy. No statistically significant difference was found in mean duration before surgery between the two groups (*p* = 0.264) (overall 65.35 ± 7.4 months). Ileal and ileocolonic involvement were the most common disease patterns, 34.0 and 33.0%, respectively. The overall recurrence rate was 24.5%, without any statistically significant difference between the two groups [10/47 (21.3%) vs. 13/47 (27.7%), *p* = 0.472]. Likewise, there were no statistically significant differences in age, gender, BMI, ASA score, duration from CD diagnosis to surgery, Montreal classification, and pre-operative medical history (Table 1).

### 3.3. Nutritional condition

The overall mean duration of nutritional treatment was 124 ± 22 days. All patients completed at least 3 months of nutritional support (range 93–475 days). Among all 47 patients who received nutritional support, 32 patients (68.1%) received PEN, 8 patients (17.0%) received EEN, 1 patient (2.1%) received EEN + PN, 6 patients (12.8%) received only dietary recommendation with micronutrient supplement. All 47 patients in the nutritional group demonstrated significant laboratory improvement of nutritional indices. Changes in inflammation and nutritional parameters are presented in Table 2. Mean-while, the nutritional group had a higher PG-SGA score, PG-SGA classification, CDAI index, and PNI (*p* < 0.0001). No statistically significant difference in BMI was observed (Table 3).

**TABLE 2:** Laboratorial inflammation and nutritional parameters.

Blood test	Nutrition (*n* = 47)	Non-nutrition (*n* = 47)	*P*-value
WBC, ×10^9^/L, mean (SD)	5.45 (± 2.45)	7.93 (± 5.25)	**0.004**
TCL, ×10^9^/L, mean (SD)	1.33 (± 0.55)	1.03 (± 0.78)	**0.035**
CRP, mg/L, mean (SD)	4.00 (1.05–10.50)	21.75 (1.00–52.25)	**0.014**
PNI	43.84 (± 7.70)	36.70 (± 7.99)	**0.000**
ESR, mm/60 min, mean (SD)	12.46 (± 2.27)	19.50 (± 4.21)	0.113
Pre-ALB, mg/L, mean (SD)	218.28 (± 73.16)	160.96 (± 69.51)	**0.000**
Alb, g/L, mean (SD)	37.32 (± 7.74)	31.66 (± 6.87)	**0.000**
Hb, g/L, mean (SD)	118.98 (± 24.56)	112.73 (± 26.03)	0.248
Fe, mmol/L, mean (SD)	10.84 (± 6.43)	9.07 (± 5.06)	0.442
Ca, mmol/L, median (range)	2.27 (± 0.27)	2.11 (± 0.16)	**0.001**
P, mmol/L, median (range)	1.25 (± 0.25)	1.12 (± 0.42)	0.083
Mg, mmol/L, mean (SD)	0.83 (± 0.07)	0.83 (± 0.05)	0.962
Folate, nmol/L, mean (SD)	19.25 (± 5.63)	12.02 (± 8.22)	**0.026**
Ferritin, ng/ml, mean (SD)	182.72 (± 75.72)	129.69 (± 49.17)	0.572
25-OH-D, ng/ml, mean (SD)	49.60 (± 20.75)	31.02 (± 11.65)	**0.024**

The bold values represent *p* < 0.05.

**TABLE 3:** Nutritional condition.

Nutritional status	Nutrition (*n* = 47)	Non-nutrition (*n* = 47)	*P*-value
BMI, kg/m^2^, mean (SD)	20.01 (± 3.15)	19.09 (± 3.43)	0.180
CDAI, mean (SD)	229.70 (± 86.43)	386.60 (± 44.16)	**0.001**
PG-SGA, mean (SD)	5.51 (± 4.41)	9.34 (± 4.11)	**0.000**
PG-SGA Classification, *n* (%)			**0.000**
A	5 (10.6)	1 (2.1)	
B	36 (76.6)	20 (42.6)	
C	6 (12.8)	26 (55.3)	
Duration of nutritional treatment, days, mean (SD)	124 (± 22)	/	
PEN, *n* (%)	32 (68.1%)	/	
EEN, *n* (%)	8 (17.0%)	/	
EEN + PN, *n* (%)	1 (2.1%)	/	
Micronutrient supplement only, *n* (%)	6 (12.8%)	/	
Post-operative ileus recovery, days, mean (SD)	2.9 (± 1.2)	3.7 (± 1.5)	0.013
Time to liquid food, days, median (range)	3.0 (2.0–4.0)	3.0 (2.0–5.0)	0.309
LOS, days, mean (SD)	20.6 (± 13.0)	14.6 (± 8.6)	**0.010**
Post-operative LOS, days, mean (SD)	8.9 (± 6.0)	13.7 (± 9.9)	**0.005**

The bold values represent *p* < 0.05.

### 3.4. Operative data

The proportion of laparoscopic surgery and emergency surgery was statistically significantly higher in the nutritional support group: 91.5 vs. 40.4% (*p* = 0.001), 7.4 vs. 31.9% (*p* < 0.0001), respectively. Fewer patients received staged surgery in the nutritional group [5 (10.7%) vs. 18 (38.3%) (*p* = 0.002)]. The proportion of patients who had resections and/or protective diversion are indicated in Table 4.

**TABLE 4:** Operative data.

Surgical index	ALL (*n* = 94)	Nutrition (*n* = 47)	Non-nutrition (*n* = 47)	*P*-value
Surgical approach, *n* (%)				**0.000**
Laparoscopy	62 (66.0)	43 (91.5)	19 (40.4)	
Laparotomy	32 (34.0)	4 (8.5)	28 (59.6)	
Type of surgery, *n* (%)				**0.000**
Emergency surgery	57 (60.6)	17 (18.1)	40 (42.6)	
Elective surgery	37 (39.4)	30 (31.9)	7 (7.4)	
Non-stage surgery, *n* (%)	71 (75.5)	42 (89.3)	29 (61.7)	**0.002**
Staged surgery, *n* (%)	23 (24.5)	5 (10.7)	18 (38.3)	
Diversion, *n* (%)	25 (26.6)	19 (40.4)	6 (12.8)	**0.002**

The bold values represent *p* < 0.05.

### 3.5. Post-operative complications and perioperative characters

No data were missing for the primary endpoint (CCI), complications, LOS, or rate of re-operation. Data regarding post-operative outcomes are presented in Table 4. A lower 30-day CCI score of nutritional group was found in the primary outcome measure (2.86 vs. 9.89, *P* = 0.015). Post-operative complications developed in 21 (22.3%) patients, 6 (12.8%) in the nutritional group and 15 (31.9%) in the non-nutritional group (*p* = 0.026). A total of 26 complication events were observed while 5 (19.2%) events of Clavien-Dindo I complications, 3 (11.5%) events of Clavien-Dindo II complications, 12 (46.2%) events of Clavien-Dindo IIIa complications, 2 (7.7%) events of Clavien-Dindo IIIb complications, and 4 (15.4%) events of Clavien-Dindo IVa complications. The common complications in our hospital were wound infection (19.2%), early post-operative bowel obstruction (19.2%), intra-abdominal abscess (19.2%), anastomotic leakage (11.5%), and excessive fluid losses of stoma (11.5%). Among all these post-operative complications, a lower incidence rate of intra-abdominal abscess (0 vs. 10.6%, *P* = 0.022) and excessive fluid losses of stoma (0 vs. 10.6%, *P* = 0.022) in nutritional group were mentioned. While no difference was observed between two groups of wound infection, early post-operative bowel obstruction, or anastomosis related complications (8.5 vs. 2.1%, *P* = 0.168, 6.4 vs. 8.5%, *P* = 0.694, 4.3 vs. 2.1%, *P* = 0.557, and 0.0 vs. 2.1%, *P* = 0.315, respectively) (Table 5). The number of multiple complication events for one patient in nutritional and non-nutritional group was 5 (10.6%) and 0 (0.0%), respectively (Table 5). Mean time of return of bowel movement, of total LOS, and of post-operative LOS were significantly fewer in the nutritional group with 2.94 ± 1.24 days, 14.62 ± 8.57 days, and 8.85 ± 6.04 days, vs. 3.67 ± 1.52 days, 20.62 ± 12.98 days, and 13.70 ± 9.90 days in the non-nutritional group (*p* = 0.013, 0.010, and 0.005, respectively) (Table 3).

**TABLE 5:** Post-operative complications and perioperative characters.

Post-operative outcome	Nutrition (*n* = 47)	Non-nutrition (*n* = 47)	*P*-value
CCI			
Mean (SD)	2.86 (± 1.15)	9.89 (± 2.31)	**0.008**
Median (IQR)	0 (0–19.96)	0 (0–2.42)	**0.015**
Post-operative complications, *n* (%)	6 (12.8)	15 (31.9)	**0.026**
Clavien-Dindo classification, *n* (%)			0.198
I	1 (2.1)	4 (8.5)	
II	1 (2.1)	2 (4.3)	
III	6 (12.8)	8 (17.0)	
IV	0 (0.0)	4 (15.4%)	
Wound infection, *n* (%)	4 (8.5)	1 (2.1)	0.168
Early post-operative bowel obstruction, *n* (%)	3 (6.4)	4 (8.5)	0.694
Intra-abdominal abscess, *n* (%)	0 (0.0)	5 (10.6)	**0.022**
Anastomotic leakage, *n* (%)	2 (4.3)	1 (2.1)	0.557
Anastomotic bleeding, *n* (%)	0 (0.0)	1 (2.1)	0.315
Excessive fluid losses of stoma	0 (0.0)	5 (10.6)	**0.022**
1 issue of post-operative complications	6 (12.8)	10 (21.3)	**0.029**
≥2 issue of post-operative complications	0 (0.0)	5 (10.6)	**0.029**

The bold values represent *p* < 0.05.

## 4. Discussion

Despite the advent of medicine, a huge step forward for patients with CD, leading to a rapid response and huge remission of disease. However, complications such as intestinal bleeding, intestinal obstruction, fistula, perforation, abscess still exist (5–7). In such kind of occasion, surgery is still the recommended treatment (7). Therefore, it is crucial to enhance the outcome by optimizing perioperative management. Pre-operative malnutrition has been shown to be associated with increased risk of post-operative complications and increased LOS after abdominal surgery (12, 23, 24). Factors such as nausea, vomiting, abdominal pain, and diarrhea reducing oral food intake contribute to malnutrition in CD patients (25, 26). Furthermore, medications such as glucocorticoids often reduce phosphorus, zinc, and calcium absorption and may lead to osteoporosis (17). Although several studies demonstrated improved disease activity and prolonged time to relapse following nutritional support (27, 28), the efficacy and protocol of nutritional support has not been fully clarified.

Consistent with previous findings, pre-operative nutritional optimization was recommended in CD patients with poor nutritional status to minimize post-operative complications (23, 29). In our study, patients with lower nutritional status and higher inflammatory level are related with post-operative complications in quantity and severity. Furthermore, the pre-operative nutritional status may also have impact on post-operative recovery of gastrointestinal function. Prolonged post-operative ileus and delay in recovery of the gastrointestinal function were commonly observed in non-nutritional group, leading to a longer LOS directly. Meanwhile, malnutrition will also increase the rate of emergency surgery, staged surgery, and diversion. The difference of surgical strategy was done for the reason leading to the surgical difficulty such as severe intra-abdominal adhesions, tissue edema, complete intestinal obstruction, or severe fistula, which indicates that successful pre-operative nutritional support could decrease the occurrence of complications during the course CD and reduce intra-abdominal inflammation.

In recent studies, diet appears to play an important role in disease pathogenesis (23, 24, 30, 31). Recent studies have shown that EEN can induce remission and mucosal healing (32, 33). So far, nutritional support, including dietary and EN is likely to play an important role during the treatment of CD. But few evidence support the effectiveness of EN. Furthermore, the evidence to support the interplay between nutritional status and post-operative complications is still lacking. Based on our results, a specific nutritional strategy may be able to play a pivotal role in improving immune-nutritional status and preventing post-operative complications of CD patients. The pre-operative EN was found to significantly improve the nutritional status scored by PG-SGA assessment and reduce the inflammatory response according to CDAI score. Meanwhile, improvements in level of serum albumin concentration, pre-albumin concentration, and TLC were also mentioned. The TLC indicates the immunological status of patient, which is also one of the important components of PNI score. Various studies have indicated that T lymphocytes affected by the systemic inflammatory response play an important role in the depression of innate cellular immunity of intestinal inflammation in cancer patients and found PNI an independent prognostic indicator in predicting post-operative complication (34, 35). Accompany with the significant decrease of inflammatory indicators such as WBC and CRP, our results indicated the beneficial effect of this new kind of comprehensive nutritional strategy in alleviating nutritional status and intestinal inflammation in CD patients.

Micronutrient status is also impacted by inflammatory severity and disease location, which may reduce surface area for absorption (36). The level of serum microelement may also be affected by some medicines (36). Weisshof et al. indicated that these micronutrients lead to deleterious downstream effects such as impaired immune response within the gut, and inflammation because of increased production of reactive oxygen species (37). In this study, we monitored important nutrients such as folate, 25-OH-vit D, calcium, potassium, magnesium, and phosphorus which were determined to be frequently suboptimal in patient with CD (38, 39). The statistic between two groups indicated that micronutrients supplementary play an important role in treatment of CD patients whose micronutrient intake from dietary was suboptimal.

This study is, to the best of our knowledge, the first study focusing on the application of EN on post-operative outcomes of CD patients. We used PSM for potential imbalances between groups, which confirmed the robustness of our conclusions. On account of CCI, we can quantify the post-operative complication more precisely with the application of such a comprehensive and sensitive measure. Regular follow-up consultation no longer than 3 or 6 months and weekly self-reporting was executed in our program since compliance is often limited for the strategy of dietary, EEN, PEN. In light of these design considerations, we believe that our results contribute new evidence for the role of nutritional support in CD patients who are candidates for surgical treatment.

Our result should be considered with some limitations. Groups were not balanced at baseline for age and ASA scores at first, however, our analyses were adjusted for these potential confounders. There was a considerable rate of missing data for PG-SGA score, CDAI index as a result of missing follow-up consultations. These missing data were handled with multiple imputation to reduce the risk of attrition bias. And the self-reporting database such as dietary compliance might be subject to reporting bias. Last but not least, this is a retrospective observational study which outcomes might be influenced by our local experience, a multi-center prospective study is excepted.

## 5. Conclusion

In conclusion, pre-operative nutritional status is correlated with post-operative outcomes while EN plays a positive role in preventing the post-operative complications. EN is a useful method for improving the pre-operative nutritional status and reducing the post-operative adverse events for CD patients undergoing surgery.

## Data availability statement

The raw data supporting the conclusions of this article will be made available by the authors, without undue reservation.

## Ethics statement

The studies involving human participants were reviewed and approved by the Ethics Committee of Shanghai Ruijin Hospital. The patients/participants provided their written informed consent to participate in this study. Written informed consent was obtained from the individual(s) for the publication of any potentially identifiable images or data included in this article.

## Author contributions

ZH, TJ, YJ, and QJ: conceptualization. TJ, YJ, and QJ: writing—original draft. ZH, MZ, AF, and YS: review and editing. SX: supervision. All authors read and agreed to the published version of the manuscript.

## Footnotes

1
http://www.assessurgery.com
